# The role of integrins in the pathogenesis of inflammatory bowel disease: Approved and investigational anti‐integrin therapies

**DOI:** 10.1002/med.21601

**Published:** 2019-06-19

**Authors:** Iris Dotan, Matthieu Allez, Silvio Danese, Mary Keir, Swati Tole, Jacqueline McBride

**Affiliations:** ^1^ Division of Gastroenterology, Rabin Medical Center, Petah Tikva, Sackler Faculty of Medicine Tel Aviv University Tel Aviv Israel; ^2^ Department of Gastroenterology, Hôpital Saint‐Louis, AP‐HP, INSERM U1160 University Denis Diderot Paris France; ^3^ Gastrointestinal Immunopathology Laboratory and IBD Unit Humanitas Clinical and Research Center Milan Italy; ^4^ Department of Research and Early Development Genentech South San Francisco California; ^5^ Department of Product Development Genentech South San Francisco California

**Keywords:** anti‐integrin therapy, Crohn's disease, ulcerative colitis, inflammatory bowel disease, integrins

## Abstract

Inflammatory bowel disease (IBD) is characterized by uncontrolled inflammation in the gastrointestinal tract. The underlying pathobiology of IBD includes an increase in infiltrating gut‐homing lymphocytes. Although lymphocyte homing is typically a tightly regulated and stepwise process involving multiple integrins and adhesion molecules expressed on endothelial cells, the distinct roles of integrin‐expressing immune cells is not fully understood in the pathology of IBD. In this review, we detail the involvement of integrins expressed on specific lymphocyte subsets in the pathogenesis of IBD and discuss the current status of approved and investigational integrin‐targeted therapies.

## 1 INTRODUCTION

Inflammatory bowel diseases (IBD) including ulcerative colitis (UC) and Crohn's disease (CD) are characterized by chronic inflammation of the gastrointestinal (GI) tract. The pathobiology of IBD involves epithelial damage, microbial dysbiosis, aberrant lymphocyte activation, infiltrates of innate immune cells, such as neutrophils, and heightened expression of pro‐ and anti‐inflammatory cytokines.^1^, ^2^, ^3^ Collectively, these diseases can be progressive and difficult to manage in a clinical setting. Current therapies for IBD are focused on alleviating symptoms and inducing and maintaining mucosal healing and clinical remission to restore patients' quality of life.^4^, ^5^ In addition to conventional therapy, including mesalamine, steroids, and immunomodulators, current therapies include biologics and small molecules that target specific molecules or disease processes.^6^ One of the major processes targeted is leukocyte recruitment to the intestinal lamina propria. In this review, we explore the most recent findings on the molecules involved in leukocyte recruitment, the distinct roles of integrin‐expressing immune cells in IBD, and the various approved and investigational integrin therapies.

### 1.1 Integrins overview

Integrins are cell surface glycoprotein receptors that play a role in leukocyte adhesion, signaling, proliferation, and migration.^7^ They are composed of heterodimeric, noncovalently interacting α and β subunits that bind to components of cell adhesion molecules (CAMs) and the extracellular matrix. Integrins exist in a low‐affinity state and must first be activated to mediate firm adhesion.^8^ Conformational changes of integrins triggered by external stimuli, such as cytokines cause a change to an open position, which enhances avidity for their respective ligands; integrins can then serve as cellular keys to direct lymphocyte migration into specific target tissues.^9^, ^10^ One example is the chemokine CCL25, which is known to activate α4β7 and is preferentially expressed in the small intestine where it interacts with lymphocytes expressing its receptor, CCR9.^11^, ^12^ The binding of integrins to tissue‐specific CAMs and the subsequent extravasation and retention of lymphocytes in peripheral tissue, including the gut, is a tightly regulated and specific process governed by such mechanisms.^13^

Most effector T lymphocytes (T_eff_) express LFA‐1 (αLβ2), which mediates binding to its ligand, intercellular adhesion molecule 1 (ICAM‐1), on high endothelial venules (HEV), such as those found in the secondary lymphoid organs including lymph nodes and Peyer's patches.^10^ The interaction between LFA‐1 and ICAM‐1 is important for tethering and T lymphocyte arrest, a prelude for transmigration to inflamed tissues. Similarly, integrin α4β1 (also known as VLA‐4) and vascular cell adhesion molecule 1 (VCAM‐1) can also direct lymphocyte trafficking to intestinal and non‐intestinal tissues.^14^ Migration to certain tissues can also be directed by additional tissue‐specific integrins, such as the directed homing of lymphocytes from the blood to the gut‐associated lymphoid tissues (GALT). Homing to GALT is facilitated by integrin α4β7 binding to the mucosal addressin cell adhesion molecule 1 (MAdCAM‐1).^14^, ^15^ Within the mucosa, the integrin αEβ7 is upregulated on a subset of infiltrating lymphocytes, and, via interactions with E‐cadherin, mediates lymphocyte retention at the epithelial layer.^16^, ^17^, ^18^

## 2 INTEGRINS AND LIGANDS IN T LYMPHOCYTE INTESTINAL HOMING

### 2.1 Integrin αLβ2 (LFA‐1)

αL is an integral membrane protein that is encoded by the *ITGAL* gene and heterodimerizes with the β2 chain, encoded by *ITGB2,* to form the integrin αLβ2, also known as LFA‐1. LFA‐1 is expressed by lymphocytes and natural killer (NK) cells and interacts with ICAMs‐1 to ‐3, specifically via the αL subunit.^19^, ^20^ LFA‐1 is involved in a variety of immunologic processes including providing costimulation during signaling,^21^, ^22^ leukocyte‐endothelial cell interactions,^23^, ^24^ and T lymphocyte‐mediated cytotoxic killing.^25^, ^26^ LFA‐1 also plays an important role in the migration of lymphocytes to the mesenteric peripheral lymph nodes and tissues via firm adhesion to ICAM‐1 on the endothelium.^27^

### 2.2 Integrin α4β1 (VLA‐4)

**Figure 1:**
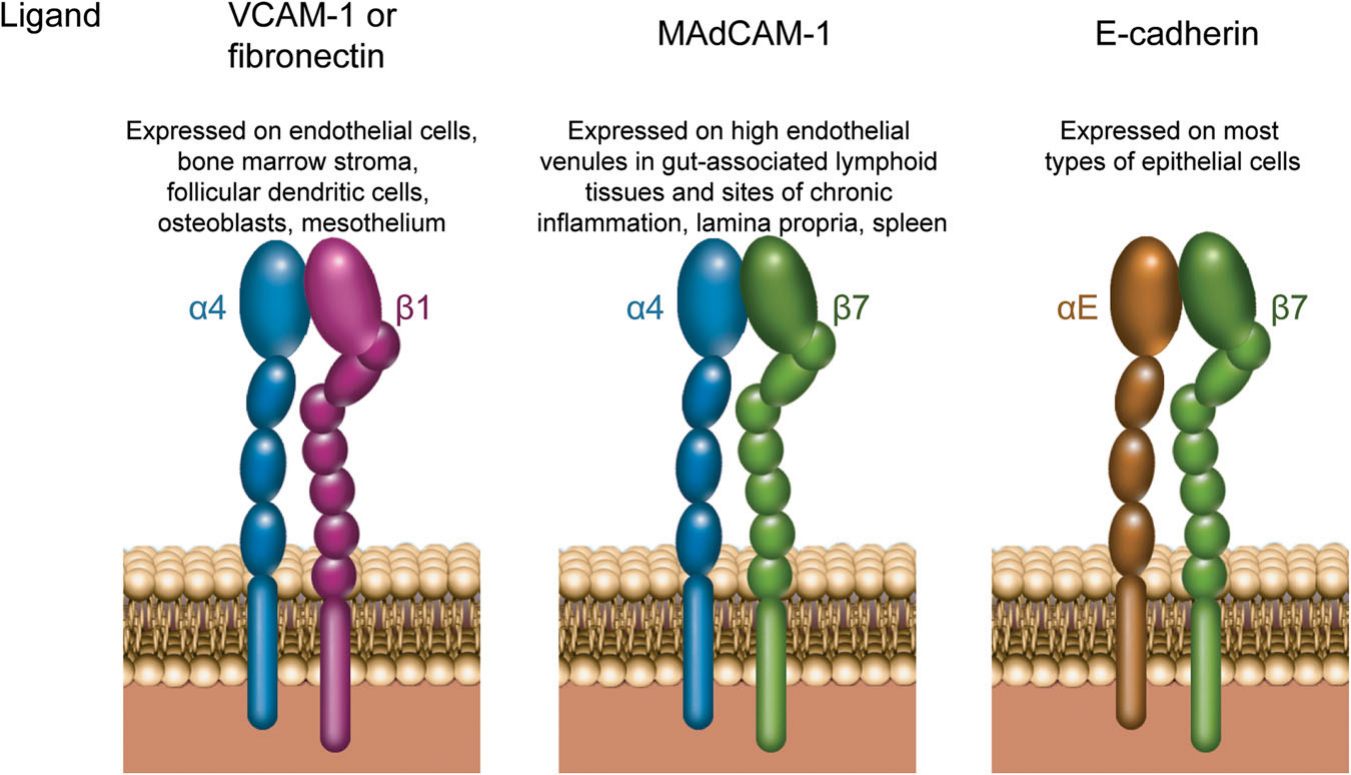
Integrin and ligand interactions. The α4β1 integrin heterodimer binds VCAM‐1 or fibronectin. The α4β7 integrin heterodimer binds MAdCAM‐1. The αEβ7 integrin heterodimer binds E‐cadherin. VCAM‐1, vascular cell adhesion molecule 1 [Color figure can be viewed at wileyonlinelibrary.com]

α4 is a transmembrane protein encoded by the *ITGA4* gene. α4 heterodimerizes with either β1 or β7 integrin, which are encoded by *ITGB1* and *ITGB7*, respectively. α4β1 interacts with VCAM‐1 (Figure 1), and its expression has been documented on most leukocytes, including, in certain circumstances, neutrophils.^28^, ^29^, ^30^ The α4β1 integrin has been shown to play a role in cell adhesion, spreading, and motility. Further, α4β1 is involved in the homing of memory and effector T lymphocytes to inflamed tissues, including intestinal and non‐intestinal tissues, such as the lung and central nervous system.^31^, ^32^

### 2.3 Integrin α4β7

The α4β7 integrin heterodimer binds MAdCAM‐1; under some circumstances, α4β7 can also bind VCAM‐1.^33^, ^34^, ^35^, ^36^ The expression of α4β7 is restricted to lymphocytes, NK cells, mast cells, basophils, and monocytes^37^, ^38^, ^39^, ^40^ and is abundant in circulating lymphocytes.^41^, ^42^ Activation of α4β7 on leukocytes results in firm adhesion to endothelial MAdCAM‐1 and then transendothelial migration of cells.^38^, ^40^ The expression of α4β7 is high on intestinal‐homing T lymphocytes, whereas the majority of T lymphocytes that circulate to non‐mucosal tissues lack expression of β7 integrin.^39^, ^41^, ^43^ A study in mice also demonstrated that α4β7 expression on T lymphocytes may be negatively regulated by expression of the β1 integrin and thus, changes in the expression of β1 may regulate the extent of intestinal homing of α4β7^+^ T lymphocytes by suppression of α4β7 expression.^44^

### 2.4 Integrin αEβ7 (CD103)

αE integrin (also known as CD103) is a transmembrane protein that is encoded on chromosome 17 by the *ITGAE* gene. The expression of αE integrin has been documented on intraepithelial T lymphocytes,^45^ dendritic cells (DCs), mast cells,^38^ innate lymphoid cells,^46^ and tumor‐infiltrating NK cells.^47^ αE integrin is only known to heterodimerize with the β7 integrin, with β7 being critical for the binding of αEβ7 on the cell surface to its ligand, E‐cadherin (Figure 1).^16^, ^48^ Of note, only ~1% of circulating lymphocytes in human peripheral blood express αEβ7 integrin, with the greatest expression on CD8^+^ lymphocytes and relatively low levels on CD4^+^ lymphocytes.^49^, ^50^

After entry into the gut, the expression of αEβ7 can be induced on the surface of T lymphocytes^51^, ^52^ and is generally thought to be induced specifically by local tumor growth factor (TGF)‐β.^17^, ^53^, ^54^, ^55^ This, in turn, allows lymphocytes to engage and embed within the epithelium as intraepithelial lymphocytes (IELs) and leads to their retention in the epithelial layer of the intestinal lumen.^16^, ^17^, ^18^ Indeed, more than 90% of IELs and approximately 40% of T lymphocytes in the lamina propria of the intestine express αEβ7.^16^, ^50^

## 3 IMMUNE CELL FUNCTIONS IN THE GUT AND IBD

There are many different subsets of T lymphocytes that modulate adaptive immune responses in the gut. Regulatory T lymphocytes (T_reg_) regulate immune responses and modulate the expansion of select T lymphocyte populations.^56^ Previous studies have noted that functional deficits in T_regs_ may potentiate inflammation by upsetting the balance between T_regs_ and T effector cells (T_effs_).^56^, ^57^, ^58^, ^59^ Memory T lymphocytes (T_mem_) rapidly proliferate to large numbers of T_effs_ upon re‐exposure to recall antigens. In both humans and mice, T_mem_ and T_eff_ cells that preferentially home to mucosal lymph nodes and tissues mediate immunity to mucosal‐specific antigens.^41^, ^60^, ^61^ Recently, the plasticity of intestinal T lymphocytes, in particular CD4^+^ T lymphocytes, has been increasingly recognized as an important factor maintaining the balance of tolerance and inflammation.^62^

**Figure 2:**
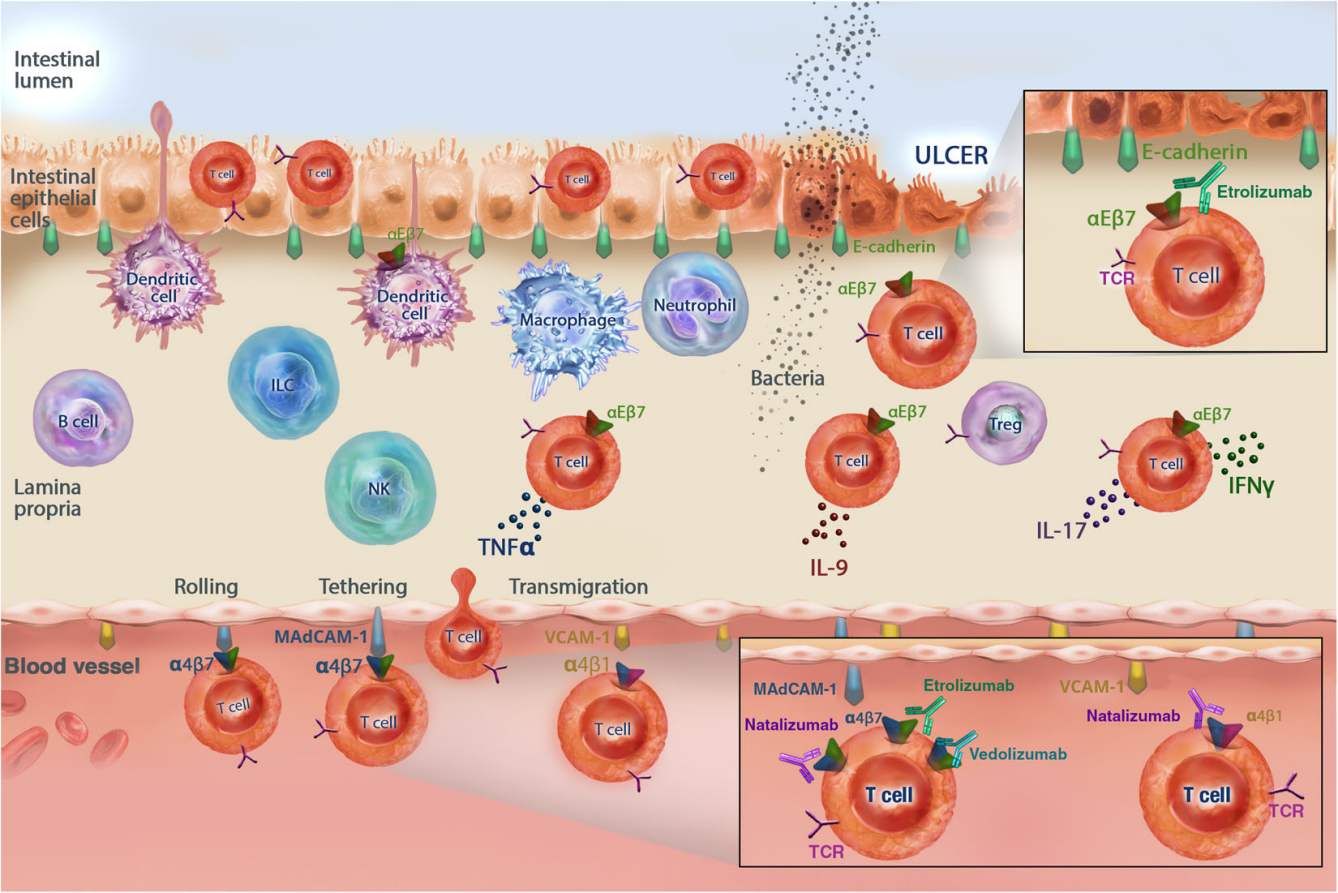
The role of integrins and immune cells in the pathogenesis of IBD. Compromised epithelial barrier function leads to increased exposure of the immune system to gut microbiota, resulting in aberrant and chronic activation of innate and adaptive immunity within the gut. In the context of an inflammatory response, DCs take up antigen and then migrate to lymph nodes where they prime antigen‐specific T lymphocytes. Activated T lymphocytes migrate from draining lymph nodes and Peyer's patches to the intestinal vasculature. Through interactions between α4β7:MAdCAM‐1 or α4β1:VCAM‐1, migration of effector T lymphocytes to the inflamed gut contributes to the local production of pro‐inflammatory mediators including IFN‐γ, IL‐6, IL‐9, and IL‐17. Once in the lamina propria, T lymphocytes may be retained through increased interactions between surface αEβ7 and E‐cadherin. α4, α4β7, αEβ7, and MAdCAM‐1 are currently targeted by integrin‐specific antibodies. Natalizumab targets the α4 integrin and thus inhibits the interaction of α4β1 or α4β7 with VCAM‐1 or MAdCAM‐1, respectively. Vedolizumab targets the α4β7 subunit and inhibits its interactions with MAdCAM‐1. Etrolizumab targets the β7 integrin and inhibits the interaction of α4β7 and αEβ7 with MAdCAM‐1 or E‐cadherin, respectively. DCs, dendritic cells; IBD, inflammatory bowel disease; MAdCAM‐1, mucosal addressin cell adhesion molecule 1 [Color figure can be viewed at wileyonlinelibrary.com]

DCs play a key role in antigen presentation and both priming and activation of T lymphocytes.^63^ DCs are sentinels surveying peripheral tissues, such as the intestine, and home to draining lymph nodes where they engage with T lymphocytes. T lymphocytes that recognize the antigen displayed by DCs in draining lymph nodes without further costimulatory cues remain inactive, or tolerant. In the context of an inflammatory response, DCs further upregulate costimulatory molecules and provide additional signals necessary to prime antigen‐specific T lymphocytes.^63^ Activated T lymphocytes then migrate into tissues to mount an immune response and become long‐lived memory populations. Given the role of both T lymphocytes and DCs in maintaining immune homeostasis in the intestine, the involvement of integrins expressed on the surface of T lymphocytes and/or DCs is highlighted in Figure 2 and discussed in detail below.

### 3.1 α4‐Expressing T lymphocytes

Early studies have described the importance of α4β7 integrin as a pivotal mediator of leukocyte infiltration into the GI tract through interaction with MAdCAM‐1 expressed on HEV within vessels of mucosal tissue.^14^, ^37^, ^41^, ^64^ Although studied extensively in the past few decades, investigations into the role of α4^+^ T lymphocytes have been hampered by the embryonic lethality of mice that carry a homozygous deletion of the α4 gene (ITGA4‐deficient).^65^ A conditional knockout mouse with T lymphocyte‐specific α4 deficiency was developed that may allow for further studies of T‐cell homing.^66^ It has been shown in preclinical models that α4β7^high^ T lymphocytes are phenotypically similar to T_eff_ memory cells in humans and preferentially home to MAdCAM‐1–rich mucosal lymph nodes and tissues,^41^, ^60^ where they contribute to immunity towards mucosal‐derived antigens.^61^

Although α4β1 and α4β7 are both expressed in humans at low levels on naive T lymphocytes,^43^ CD4^+^ T_mem_ lymphocytes can express high levels of either α4β1 or α4β7 integrin, or both integrins.^39^, ^41^, ^43^ α4β1:VCAM may be able to drive intestinal homing independently of α4β7/MAdCAM under some conditions.^67^ The majority of gut homing is likely facilitated through the interaction between α4β7 integrin and MAdCAM‐1, which is constitutively expressed in intestinal tissue and increased in IBD.^15^, ^68^ MAdCAM‐1^+^ venules have also been described in lymphoid aggregates in the deeper layers of the intestines of patients with CD.^68^

T_reg_ cells also use α4β7 to gain entry into the gut,^69^ but there is also likely local T_reg_ differentiation and expansion.^70^ It has been demonstrated that homing of both T_reg_ and T_eff_ populations were reduced with α4β7 blockade.^69^

### 3.2 αEβ7‐Expressing T lymphocytes

The highest proportion of αEβ7‐expressing effector‐memory CD8^+^ and CD4^+^ T lymphocytes can be observed in both small intestine and colon; significant fractions also are observed in the lung and draining lymph nodes relative to other mucosal tissues.^49^, ^71^ A recent study has also shown that αE^+^ cells are more abundant in the ileum in comparison with the colon, with no association with disease activity.^72^ Furthermore, αEβ7 is expressed predominantly on large subsets of intraepithelial CD4^+^ and CD8^+^ T lymphocytes or IELs.^73^ Similarly, in the murine bowel, most intestinal intraepithelial T lymphocytes express αEβ7.^17^, ^45^ In murine studies, long‐term immunity was shown to be maintained because of the function of tissue‐resident memory T lymphocytes that are αEβ7^+^,^74^ which require αEβ7 expression to persist within the intestinal epithelium.^52^

Preclinical data support a procolitogenic role for αEβ7‐expressing T lymphocytes. In a recent study, the appearance of a subset of γδ T lymphocytes bearing αEβ7 in the mesenteric lymph node and intestine was reported to precede the development of colitis in the SAMP mouse model of ileitis.^75^ In this study, a distinct subpopulation of αEβ7^+^α4β7^high^ γδ exacerbated Th1/Th17 T‐lymphocyte accumulation in colonic tissue and disease when transferred with CD4^+^ T cells to immunodeficient RAG mice. Correspondingly, in an IL‐2–deficient mouse model of CD4‐driven colitis, administration of an antibody against αEβ7 reduced lamina propria CD4^+^ T‐lymphocyte levels and their production of interferon (IFN)‐γ.^76^

Studies in human IBD patients also support a role for αEβ7 in disease pathology. In a comparison of colonic CD4^+^ T lymphocytes in patients with UC versus healthy controls, it was shown that the αEβ7^+^ CD4^+^ T lymphocyte subset was enriched for Th17 cells and for Th17/Th1 cells—a subset which express both pro‐inflammatory cytokines IL‐17 and IFN‐γ.^73^ Dual IL‐17A/IFN‐γ–producing Th17/Th1 T lymphocytes have been described in both CD and UC and increased inflammatory potential that may play a role in disease pathology.^73^, ^77^ αEβ7 integrin expression is also highly expressed on CD8^+^ and Th9 lymphocytes.^78^ The involvement of Th9 cells in the pathobiology of IBD is increasingly appreciated as blockade of IL‐9 has been shown to attenuate disease severity in experimental models of IBD.^79^, ^80^ In agreement with a loss of regulatory function and a gain of pro‐inflammatory function for αEβ7^+^ T lymphocytes in the disease pathogenesis of UC, these cells have lower expression of T_reg_‐associated genes, including FOXP3, IL‐10, CTLA‐4, and ICOS, compared with the αEβ7^−^ T‐lymphocyte subset.^73^ Furthermore, in CD, αEβ7 is highly expressed on the expanded subset of CD4^+^ T lymphocytes expressing the activating receptor NKG2D, which exhibits pro‐inflammatory and cytotoxic properties.^81^ Recently, it was also demonstrated that the extent of CD4^+^CD69^+^αE^+^ tissue‐resident T_mem_ (TRM) cells is predictive of the development of flares in patients with IBD,^82^ and CD4 TRM cells in CD are a major source of mucosal TNF‐α.^83^

The frequency of αEβ7^+^‐bearing T lymphocytes is generally higher in the ileum relative to the colon—this, coupled with a dysregulated function of these cells, may exacerbate the widespread inflammation associated with CD.^72^ The role of αEβ7 on lymphocytes beyond maintenance of retention has yet to be elucidated and may or may not contribute directly to the cytotoxic activity of these cells at the epithelium. Studies have shown that αE‐expressing T cells are involved in the destruction of intestinal epithelial cells and may mediate localized tissue damage.^53^, ^84^ At minimum, the ability of αEβ7 to facilitate close proximity of cytotoxic CD4^+^ or CD8^+^ T lymphocytes with their target cells may be one key driver mediating immune activation.^85^

Our understanding of the role of αEβ7^+^ T lymphocytes in IBD is derived from both mouse models and human studies. However, it is important to note that there are challenges when extrapolating directly from mouse studies to understanding human IBD pathophysiology. Key differences include varied microbial community structure as well as altered localization and/or types of leukocytes in the gut,^86^, ^87^, ^88^ particularly in the phenotype of FOXP3^+^ T lymphocytes.^89^

### 3.3 DCs and other immune cells

In addition to the involvement of T lymphocytes in maintaining intestinal homeostasis, in vitro and in vivo data in both mice and humans have demonstrated that intestinal DCs orchestrate protective immune responses to antigens derived from pathogens as well as maintaining immune tolerance to commensal microbiota and food antigens through their primary role in antigen presentation to T lymphocytes. Although it is not known whether there is a role for α4 on DCs, mesenteric lymph node DCs isolated from the human gut have been shown to express αEβ7.^90^, ^91^ Studies in mice have shown that the majority of intestinal DCs express αEβ7.^92^ In mice, it has been demonstrated that αE^+^ DCs may exert tolerogenic or inflammatory functions depending on the environment.^93^ In addition, murine studies have also shown that αEβ7^+^ DCs may promote inflammation by activating CD4^+^ T lymphocytes to exhibit Th1 behavior^94^ as well as stimulate gut‐tropic CD8^+^ effector T lymphocytes.^92^ It was recently demonstrated that the numbers of αEβ7^+^ DCs in UC and CD were reduced in the inflamed mucosa in comparison with non‐IBD gut tissue.^95^, ^96^, ^97^ In addition to reduced numbers, it was reported in UC that resident αEβ7^+^ DCs showed an impaired ability to generate FOXP3^+^ T_reg_ cells but had acquired a potent ability to drive differentiation of inflammatory Th1/Th2/Th17 T_eff_ lymphocytes.^98^ In the pathogenesis of UC, it is hypothesized that these resident αEβ7^+^ DCs may have lost their ability to produce retinoic acid and, therefore, their ability to induce T_reg_ cells.^97^ These data are important, at least in UC, where typically tolerogenic αEβ7^+^ DCs may be altered into colitogenic αEβ7^+^ DCs.^98^ Future studies will be necessary to further understand the alterations of resident cells under disease conditions at the molecular level and their contribution to the pathogenesis of both UC and CD.

## 4 A MODEL FOR INTEGRINS AND T LYMPHOCYTES AS THERAPEUTIC TARGETS IN THE PATHOGENESIS OF IBD

The proposed role of T lymphocytes in the pathogenesis of IBD is illustrated in Figure 2. Compromised epithelial barrier function may be an initiating event in IBD pathobiology, with microbial exposure of the immune system resulting in downstream effects that may include: (1) aberrant and chronic activation of innate and adaptive immunity, such as macrophage and DC activation and migration of neutrophils to the inflamed gut and (2) local production of pro‐inflammatory mediators including IFN‐γ, IL‐6, IL‐9, and IL‐17.^3^ Further, VCAM‐1 and MAdCAM‐1 are increased in inflamed intestinal biopsies from CD and UC patients^15^, ^68^ and may enhance migration of cytotoxic, pro‐inflammatory T lymphocytes into the lamina propria through α4β1 and α4β7, respectively.^69^, ^99^ Once in the lamina propria, cytotoxic T lymphocytes may be retained through increased interactions between αEβ7 and E‐cadherin expressed on intestinal epithelial cells.^53^, ^100^ Chronic migration, retention of inflammatory immune cells and increased production of pro‐inflammatory mediators may perpetuate and exacerbate pathogenesis in IBD.

### 4.1 α4β1 and α4β7 as a target for IBD treatment

On the basis of the data showing that VCAM‐1 and MAdCAM‐1 are upregulated in IBD, α4 was the first integrin to be therapeutically targeted for the treatment of UC and CD (Table 1).^99^, ^101^ In preclinical studies in the cotton‐top tamarin model of colitis, it was demonstrated that treatment with a monoclonal antibody against α4—blocking both α4β1 and α4β7—led to a significant reduction in colitis compared with placebo‐treated animals.^101^ These data highlighted the potential of integrin‐mediated therapies for the treatment of IBD and led to the development of an α4 humanized antibody, natalizumab, for CD. In a double‐blind, placebo‐controlled trial, natalizumab demonstrated increased clinical response and remission rates in patients with moderate to severe CD at week 6 when compared with placebo‐treated patients.^102^ These positive results were further confirmed in subsequent phase 3 studies, which showed that natalizumab‐treated patients had higher rates of remission through week 36 versus placebo,^103^ as well as superior responses at weeks 4, 8, and 12, compared with placebo‐treated patients.^104^ However, an increased risk of fatal progressive multifocal leukoencephalopathy (PML), likely the result of impaired immune cell trafficking to the CNS, was associated with natalizumab^103^; as a result, it is rarely used for the treatment of CD.

**Table 1:** Anti‐integrin signaling therapies in UC and CD

	Molecules targeted	Class of drug	Development stage	Developing company	References
Natalizumab	α4	Anti‐integrin monoclonal antibody	In the market (limited use)	Biogen	102, 103, 104
Vedolizumab	α4β7	Anti‐integrin monoclonal antibody	In the market	Takeda	109, 110
Etrolizumab	α4β7; αEβ7	Anti‐integrin monoclonal antibody	Phase 3	Genentech	130, 131, 132, 133
AJM300	α4	Small molecule antagonist	Phase 2a	Ajinomoto Pharmaceuticals	105
Abrilumab	α4β7	Anti‐integrin monoclonal antibody	Phase 2b	Amgen, AstraZeneca	123, 144

Abbreviations: CD, Crohn's disease; UC, ulcerative colitis.

The use of small molecule α4 antagonists has continued to be of interest for the treatment of IBD. The orally active small molecule α4 antagonist AJM300 is one such agent currently in development.^105^ In a double‐blind, placebo‐controlled, phase 2a study in patients with moderately active UC, AJM300 improved all measured indices of IBD compared with placebo, including increased rates of clinical remission and mucosal healing, with no reported serious adverse events.^105^

### 4.2 Increasing specificity for the gut via targeting α4β7

Vedolizumab is a humanized antibody which binds to α4β7 and was shown to specifically inhibit the interaction between α4β*7* integrin and its ligands, MAdCAM‐1 and fibronectin (Table 1).^106^ Given that MAdCAM‐1 expression is largely restricted to the intestine, the inhibitory effects of vedolizumab are regarded as gut selective. In initial preclinical studies, the mouse monoclonal antibody ACT‐1 (from which vedolizumab was derived) improved stool consistency and reduced leukocyte infiltration in the cotton‐top tamarin model of colitis.^107^ The gut selectivity of the α4β7 integrin was further supported in studies in cynomolgus monkeys treated with vedolizumab, which demonstrated a decrease in the frequency of β7‐expressing cells in the intestine corresponding with an increase in β7^high^‐expressing cells in the peripheral blood.^108^ There were no changes in the frequency of β7^low^‐expressing cells nor any changes observed outside of the intestine.

Vedolizumab was shown to be safe and effective for the treatment of IBD in humans in phase 3 studies. The phase 3 GEMINI I study in patients with UC treated with vedolizumab demonstrated increased response rates at week 6 compared with placebo. Of these responders, more than 40% of patients maintained the response through week 52.^109^ In the GEMINI II study of patients with CD, vedolizumab treatment resulted in higher remission rates at week 6 that continued through week 52 compared with placebo‐treated patients.^110^ Conversely, in the GEMINI III trial of patients with CD who had failed therapy with a corticosteroid, immunosuppressant, or anti‐TNF (aTNF), those who were treated with vedolizumab were not more likely than placebo‐treated patients to achieve clinical remission at week 6; however, clinical remission in vedolizumab‐treated patients was observed at week 10.^111^ These data led to the approval of vedolizumab for the treatment of moderate to severe UC and CD in 2014.

Importantly, the gut selectivity of vedolizumab in humans has been demonstrated,^112^ and no cases of PML have been attributed to vedolizumab.^113^ An analysis of vedolizumab exposure up to May 19, 2016, in the general population estimated that the risk for vedolizumab‐related PML is between 0.00 and 6.75 cases per 100 000 patient years.^114^ In keeping with the anticipated rate in the general population and known risk factors of PML, a single case of PML in a patient with undiagnosed HIV and years of immunosuppressive use has been reported.^115^ The development of PML in patients treated with natalizumab is believed to be because of impaired immune surveillance of the CNS. No significant changes in T lymphocyte populations in the cerebrospinal fluid (CSF) were observed in vedolizumab‐treated healthy volunteers.^116^ In contrast to VCAM‐1, which is expressed on cerebral endothelial vessels, perivascular tissue, and meninges, the expression of MAdCAM‐1 has not been demonstrated in these tissues.^117^, ^118^, ^119^, ^120^

Fischer et al^69^ reported that in a humanized mouse model of colitis, vedolizumab inhibited the homing of UC T_eff_ cells and T_reg_ cells to the mouse colon. A subsequent publication from the same group showed that vedolizumab had only marginal effects on homing of CD T_eff_ cells to the ileum, whereas natalizumab reduced CD T_eff_ cell homing.^67^ These data suggest that inhibition of α4β7‐dependent homing of CD T_eff_ cells may be bypassed by a compensatory homing mechanism through α4β1:VCAM‐1, which is supported by the increased α4β1 expression on T_eff_ cells from patients with CD. Results from these two studies suggest that the underlying pathologic and trafficking mechanisms within CD versus UC may differ at the cellular level and could perhaps explain the trend towards greater improvements with vedolizumab treatment in patients with UC compared with CD.^67^, ^68^ The relative importance of α4β7 and α4β1 integrin expression on trafficking of pro‐inflammatory T lymphocytes between UC and CD remains to be fully elucidated.

Abrilumab (AMG181/MEDI7183) is a fully human monoclonal antibody directed against α4β7.^121^, ^122^ In a phase 2b multicenter, randomized double‐blind study in patients with moderate to severe UC who were refractory to aTNF or immunomodulator therapy, abrilumab demonstrated evidence of efficacy and an acceptable safety profile, with no reported cases of PML.^123^ Although there was some evidence for efficacy of abrilumab in CD, the primary end point, CDAI remission (score < 150) at week 8 was not met in the phase 2b, multicenter, randomized double‐blind study in patients with moderate to severe disease.^123^

PTG‐100 is an investigational oral α4β7 integrin antagonist peptide. In early 2018, the phase 2b clinical trial of PTG‐100 for patients with moderate to severe UC was discontinued when a planned interim analysis conducted by an independent data monitoring committee deemed the trial to be futile after review of unblinded data from 65 of the 240 patients who had completed 12 weeks of treatment on the basis of an analysis of the primary end point of clinical remission.^124^ A subsequent independent blinded reanalysis of the endoscopy data revealed an error by the original central reader that resulted in a higher than anticipated placebo effect, which led to the futile outcome assessment. A comprehensive rereview of the interim analysis data showed that PTG‐100 treatment did indeed show signals of clinical efficacy over placebo.^125^ No safety concerns were noted in either analysis.^126^ Further clinical studies of PTG‐100 for the treatment of IBD have been discontinued.^127^

### 4.3 Targeting both α4β7 and αEβ7 integrins for treatment of IBD

Etrolizumab is a humanized immunoglobulin (Ig)G1 monoclonal antibody that selectively targets the β7 subunit of both α4β7 and αEβ7 integrins with high affinity and blocks interactions with their respective ligands, MAdCAM‐1 and E‐cadherin (Table 1). In a humanized mouse model, gut‐specific lymphocyte trafficking to the inflamed colon was attenuated to a greater degree by etrolizumab in comparison with the anti‐α4β7 antibody vedolizumab.^100^ Similar results were also reported in an earlier mouse model treated with the anti‐β7 antibody FIB504.^128^ Furthermore, administration of an antibody against αEβ7 attenuated immunization‐induced colitis in IL‐2–deficient mice,^17^, ^76^ providing additional evidence that αEβ7 is an important player in the inflammatory processes associated with IBD pathogenesis.^89^ In addition to immune functions, a study in mice showed that β7^+^ IELs also calibrate metabolism by binding GLP‐1 and limiting its systemic availability, suggesting potential added benefit of β7 blockade.^129^

The efficacy of etrolizumab in patients with UC was demonstrated in the phase 2 EUCALYPTUS study which showed higher rates of remission at week 10 and similar frequency of adverse events compared with placebo,^130^ establishing the therapeutic potential of targeting both α4β7 and αEβ7 with anti‐integrin therapy. Furthermore, in etrolizumab‐treated patients, αEβ7^+^ cells in the intestinal epithelium were reduced in comparison with the placebo group, with no observed decrease in αEβ7^+^ cells in the lamina propria in either treatment group, indicating that binding of etrolizumab to αEβ7 cells was preventing these cells from binding E‐cadherin and being retained in the epithelium.^130^

Phase 3 studies for etrolizumab are ongoing in both UC and CD. The efficacy and safety of etrolizumab in patients with moderate to severe UC who have experienced aTNF failure is being evaluated. The data from the UC induction cohort from the HICKORY trial showed that aTNF‐intolerant or ‐refractory patients treated with etrolizumab had rapid and sustained improvements in endoscopy, rectal bleeding, stool frequency, and the relevant disease biomarkers, C‐reactive protein, and fecal calprotectin.^131^, ^132^ Additional post hoc analyses of the HICKORY induction cohort indicated that patients who showed improvement in endoscopic score also achieved higher rates of remission of rectal bleeding, lower stool frequency scores, and greater reductions in C‐reactive protein and fecal calprotectin.^131^

BERGAMOT is an ongoing, placebo‐controlled phase 3 study evaluating the efficacy of etrolizumab for the treatment of patients with moderate to severe CD who have been previously treated with immunomodulators, corticosteroids, and/or aTNFs.^133^ The 14‐week exploratory induction cohort enrolled a highly experienced patient population, with more than 70% having failed prior treatment with aTNFs. In this induction cohort, treatment with both etrolizumab 105 mg and 210 mg resulted in higher rates of clinically meaningful endoscopic improvement compared with placebo. Furthermore, symptomatic remission was reported as early as week 6 and was observed consistently through week 14.^133^ These data indicate that blockade of both α4β7^+^ and αEβ7^+^ cells may be efficacious in this difficult‐to‐treat CD population.

### 4.4 MAdCAM‐1 as a target for the treatment of IBD

In addition to anti‐integrin therapies, the anti–MAdCAM‐1 monoclonal antibody, SHP647/ontamalimab (formerly PF‐00547659), is being evaluated for the treatment of IBD. In the phase 2 TURANDOT trial in patients with moderate to severe UC, 12 weeks of SHP647/ontamalimab treatment resulted in significantly greater remission rates at 7.5 mg, 22.5 mg, and 75 mg doses every 4 weeks compared with placebo.^134^ However, the phase 2 OPERA study in patients with active refractory CD failed to meet its primary end point, despite evidence of target engagement.^135^ In the phase 1 safety study, TOSCA, in patients with active CD, 12 weeks of SHP647/ontamalimab induction therapy did not result in a reduction in CSF lymphocytes or T‐cell subsets or CD4:CD8 ratio.^136^ The data from extension studies for UC (TURNADOT II) and CD (OPERA and TOSCA) demonstrated that efficacy achieved with SHP647/ontamalimab induction were maintained for up to 144 weeks and 72 weeks, respectively.^137^, ^138^ Although SHP647/ontamalimab demonstrated a favorable safety profile in both UC and CD, its efficacy was less robust for CD, highlighting the complexity of the mechanisms underlying IBD as well as the therapeutic challenges.

## 5 SAFETY OF ANTI‐INTEGRIN THERAPIES

Although a large proportion of patients with IBD respond to corticosteroids or immunomodulators, up to 40% of IBD patients are refractory to these therapies.^139^, ^140^, ^141^ Thus, there is still an unmet medical need for safe and effective therapies for the treatment of IBD. Targeted anti‐integrin therapies offer a promising alternative for the treatment of IBD. As previously discussed, although natalizumab was associated with the development of PML, extensive evidence suggests that selectively targeting β7‐containing integrins or MAdCAM‐1 offers effective treatment of IBD with a favorable safety profile to date. Current clinical research suggests that inhibition of α4β7 via vedolizumab or other small molecules, or dual blockade of α4β7 and αEβ7 via etrolizumab should not lead to any significant side effects outside the gut, including the risk of PML.^142^ In terms of α4β7 blockade, although it has been shown that homing of both T_eff_ and T_reg_ cells can be impacted,^69^ clinical studies do not support the idea that β7 blockade may lead to any worsening of inflammation. This indicates that in the context of the disease the relative proportions of these populations may be more relevant and the mechanisms of T_reg_ homing and expansion are less understood.

αEβ7 expression is not restricted to the gut, and αEβ7‐expressing immune cells are also found in non‐intestinal tissues, including lung, skin, liver, and spleen,^49^ although their role in these organs is less clear. The impact of blockade of αEβ7 expressing cells is unknown. Completed phase 1 and 2 and ongoing phase 3 clinical trials have shown that blockade of αEβ7 with etrolizumab is well tolerated, with rates of serious adverse events similar to those with placebo.^130^, ^131^, ^132^, ^133^ In addition, both CD4^+^αEβ7^+^ and CD8^+^αEβ7^+^cells are pro‐inflammatory in phenotype; CD4^+^αEβ7^+^ demonstrating fewer markers associated with T_reg_ cells including FoxP3 relative to CD4^+^αEβ7^−^ lymphocytes.^73^, ^82^, ^143^

## 6 CONCLUSIONS AND FUTURE DIRECTIONS

Evidence suggests that integrins are critical players in IBD pathogenesis, and recent clinical data have begun to elucidate the therapeutic benefit of anti‐integrin blockade in IBD. Anti‐integrin therapies with gut selectivity offer a new class of therapeutics that are safe and well tolerated and hold significant promise for efficacy in both UC and CD. Ongoing clinical trials of novel therapeutic agents targeting integrin‐mediated intestinal homing will generate substantial data to further our understanding of the key players and processes in IBD.

## AUTHOR CONTRIBUTIONS

All authors were members of the writing group and participated in the development of the report, agreed on the content, reviewed drafts, and approved the final version.

## DISCLOSURES

Iris Dotan served as a speaker, consultant, and/or advisory board member for Genentech, Abbvie, Pfizer, Janssen, Takeda, Ferring, Roche, Rafa Laboratories, Falk Pharma, Given Imaging, Protalix, Medtronix, Celltrion, and Neopharm. Matthieu Allez received honoraria from Abbvie, MSD, Janssen, Takeda, Pfizer, Celgene, Roche/Genentech, Novartis, Ferring, Tillots, Mayoli, and UCB. Silvio Danese served as a speaker, consultant, and/or advisory board member for Abbvie, Ferring, Hospira, Johnson and Johnson, Janssen, Merck, MSD, Takeda, Mundipharma, Pfizer Inc, Tigenix, UCB Pharma, Vifor, Biogen, Celgene, Allergan, Celltrion, Sandoz, and Boehringer Ingelheim. Mary Keir, Jacqueline McBride, and Swati Tole are all employees of Genentech, a member of the Roche Group.
